# Ab Initio Study of Ferroelectric Critical Size of SnTe Low-Dimensional Nanostructures

**DOI:** 10.3390/nano10040732

**Published:** 2020-04-11

**Authors:** Takahiro Shimada, Koichiro Minaguro, Tao Xu, Jie Wang, Takayuki Kitamura

**Affiliations:** 1 Department of Mechanical Engineering and Science, Kyoto University, Nishikyo-ku, Kyoto 615-8540, Japan; minaguro.koichiro.77w@st.kyoto-u.ac.jp (K.M.); takayuki.kitamura.kyoto@gmail.com (T.K.); 2 Materials Genome Institute, Shanghai University, Shanghai Materials Genome Institute, Shanghai 200444, China; xutao6313@shu.edu.cn; 3 Department of Engineering Mechanics & Key Laboratory of Soft Machines and Smart Devices of Zhejiang Province, School of Aeronautics and Astronautics, Zhejiang University, Hangzhou 310027, China; jw@zju.edu.cn

**Keywords:** ferroelectricity, SnTe, nanoribbon, nanoflakes, critical size, density-functional theory

## Abstract

Beyond a ferroelectric critical thickness of several nanometers existed in conventional ferroelectric perovskite oxides, ferroelectricity in ultimately thin dimensions was recently discovered in SnTe monolayers. This discovery suggests the possibility that SnTe can sustain ferroelectricity during further low-dimensional miniaturization. Here, we investigate a ferroelectric critical size of low-dimensional SnTe nanostructures such as nanoribbons (1D) and nanoflakes (0D) using first-principle density-functional theory calculations. We demonstrate that the smallest (one-unit-cell width) SnTe nanoribbon can sustain ferroelectricity and there is no ferroelectric critical size in the SnTe nanoribbons. On the other hand, the SnTe nanoflakes form a vortex of polarization and lose their toroidal ferroelectricity below the surface area of 4 × 4 unit cells (about 25 Å on one side). We also reveal the atomic and electronic mechanism of the absence or presence of critical size in SnTe low-dimensional nanostructures. Our result provides an insight into intrinsic ferroelectric critical size for low-dimensional chalcogenide layered materials.

## 1. Introduction

Ferroelectrics exhibit spontaneous polarization that can be reversed by an external electric field, due to their noncentrosymmetric crystal structure having a relative displacement of cations and anions in a ferroelectric (FE) phase. Ferroelectric properties have attracted attention due to their technological applications such as ferroelectric memory (FeRAM), sensors, MEMS/NEMS, and actuators [1,2,3]. To enhance the performance of these devices, it is necessary to reduce the size of ferroelectrics and integrate them at a high density. In recent years, with the progress of manufacturing technology, nanoscale ferroelectric materials with low-dimensional structures such as nano-thin films [4,5] (two-dimensional; 2D), nanowires [6,7], nanotubes [8,9] (one-dimensional; 1D), and nanodots [10,11] (zero-dimensional; 0D) have been synthesized for the high-performance, high-integration of nano-devices.

However, ferroelectricity disappears when the size of the ferroelectric material becomes nanoscale (ferroelectric critical size): in perovskite oxide PbTiO_3_ and BaTiO_3_ nanofilms, ferroelectricity disappears when the thickness of the films becomes 2 nm or less [12,13,14,15]. The appearance of ferroelectric critical size was explained by two aspects: (I) effect of electrostatic (depolarization) field and (II) the reconstruction and rearrangement of atomic and electronic structure at surfaces or edges. At the surface of ferroelectric materials, the surface polarization charge is formed due to the termination of polarization, and the depolarization field formed in the opposite direction of spontaneous polarization suppresses the ferroelectricity [16]. In particular, when the material dimensions become nanoscale, the ratio of the surface or edge to the entire volume increases, the influence of the depolarization field formed by the surface charge becomes dominant, and the ferroelectricity of the entire material disappears. This is factor (I). In general, ferroelectricity originates from a delicate balance between long-range interaction due to Coulomb force, which is the driving force for the relative displacement (ferroelectric displacement) of cations and anions in the crystal, and short-range interaction due to covalent bonds that stabilize the centrosymmetric structure [17]. In nanoscale materials, the long-range interaction is reduced due to the absence of atoms outside of material surfaces, and thereby the balance between long-range and short-range interaction is broken. In particular, such interactions are also changed due to the reconstruction and rearrangement of atomic and electronic structures at surfaces or edges. This is factor (II). For these reasons, the ferroelectric critical size appears. This physical limitation prevents the miniaturization of ferroelectric materials beyond the critical size.

In recent years, however, ferroelectricity was discovered in the monolayer structure of chalcogenide SnTe in the in-plane direction [18]. This indicates that ferroelectricity can exist in a structure with an atomic thickness. Obviously, this discovery is beyond the long-believed ferroelectric critical thickness of several nanometers. Since the nanostructure is commonly utilized in a low-dimensional form, it is scientifically interesting and technologically important to investigate whether ferroelectricity is also sustained in ultimate SnTe nanoribbons (1D) and SnTe nanoflakes (0D) in addition to the discovered monolayer (2D) form. However, the ferroelectric critical size for SnTe nanoribbons and nanoflakes has not yet been reported.

In this study, we investigate whether a ferroelectric critical size exists in low-dimensional structure of SnTe, the nanoribbons (1D) and nanoflakes (0D) using first-principle, density-functional theory (DFT) calculations.

## 2. Materials and Methods 

**Figure 1:**
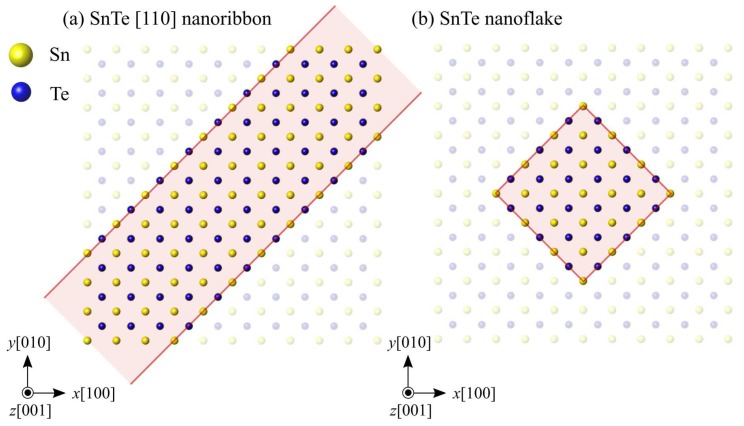
Schematic illustration of (**a**) SnTe nanoribbon and (**b**) SnTe nanoflake.

We focus on SnTe nanoribbons and nanoflakes with an edge structure. SnTe has two types of edges formed along the [110] and [100] directions. Henceforth, these edge structures are called [110] edge and [100] edge, respectively. Table 1 shows the preliminarily calculated formation energies of the [110] and [100] edges. Here, the edge formation energy is calculated by *E*_edge_ = (*E*_nanoribbon_ – *E*_monolayer_)/2*l*, where *E*_nanoribbon_ and *E*_monolayer_ are the total energies of SnTe nanoribbons and SnTe monolayer, respectively, and *l* is the length of edge in the nanoribbon model, shown later. The formation energy of the [110] edge is lower, and thus more stable, than that of the [100] edge. In addition, the [110] edge structure was experimentally observed at the edge of the SnTe monolayers [18,19,20]. Following these experimental and theoretical results, we thus analyze the nanoribbons and nanoflakes consisting of the [110] edges, as shown in Figure 1. Figure 1 shows the paraelectric phase of SnTe monolayer with a space group of *Fm3m*. In the ferroelectric phase, Sn and Te atoms are spontaneously displaced along the [110] direction. The space group of the ferroelectric SnTe monolayer is *Pmn*2_1_. The following SnTe nanoribbons and nanoflakes are in the ferroelectric phase, and thus modeled with a small initial displacement along [110]. Note that the electronic origin of ferroelectricity and alternating short and long bonds has already been discussed by Liu et al. [21], and they revealed that the stabilization of the ferroelectric phase and large distortion originates from an interplay between hybridization interactions of Sn-Te, which act as a driving force for the ferroelectricity, and Pauli repulsions, which tend to suppress the ferroelectricity.

**Figure 2:**
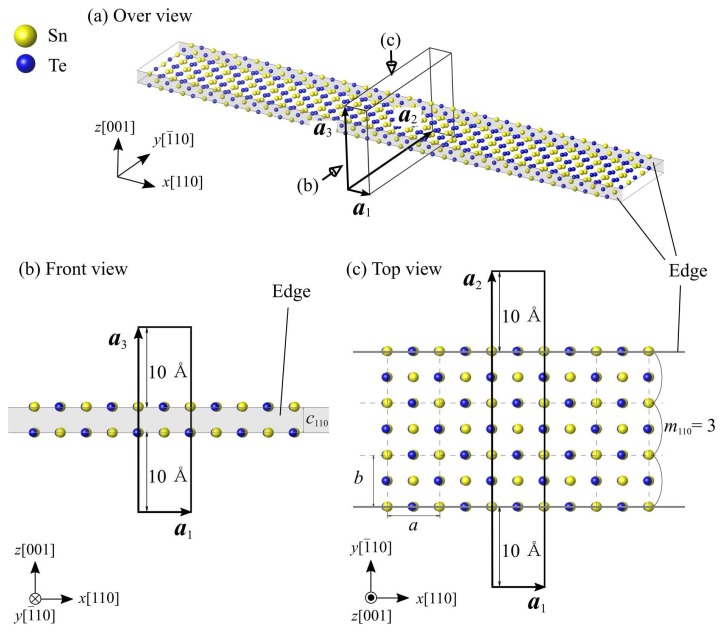
Simulation models of SnTe nanoribbon with 3-unit-cells width. The gray area and solid lines indicate edges of SnTe nanoribbon. The solid boxes represent the simulation cells. ***a***_1_, ***a***_2_ and ***a***_3_ indicate the simulation cell vectors.

Figure 2 shows the simulation model of the SnTe nanoribbons. Here, *m*_110_ denotes the number of unit cells constituting the nanoribbon width. To explore the critical ferroelectric size, we calculate several SnTe nanoribbons with different widths of *m*_110_ = 1 to 10 (6 to 65 Å). The model of a nanoribbon width 3-unit-cells width (*m*_110_ = 3) is shown in Figure 2 as an example. In this simulation model, there are 4*m*_110_ + 2 Sn and Te atoms each, for a total of 8*m*_110_ + 4 atoms. The three-dimensional periodic boundary condition is applied to the simulation cell. To prevent undesirable interactions between the nanoribbons in the neighboring image cells, a vacuum region of *l*_v_ = 20 Å in the *y*- and *z* directions. ***a***_1_, ***a***_2_, and ***a***_3_ in the figure are the simulation cell vectors, and are represented by

***a***_1_ = (*a*, 0, 0), (1)

***a***_2_ = (0, *m*_110_*b + l*_v_, 0), (2)

***a***_3_ = (0, 0, *c + l*_v_), (3)

where *a*, *b*, and *c* are the equilibrium lattice constants of the SnTe monolayer, *a* = 6.520 Å, *b* = 6.479 Å, and *c* = 3.240 Å.

**Figure 3:**
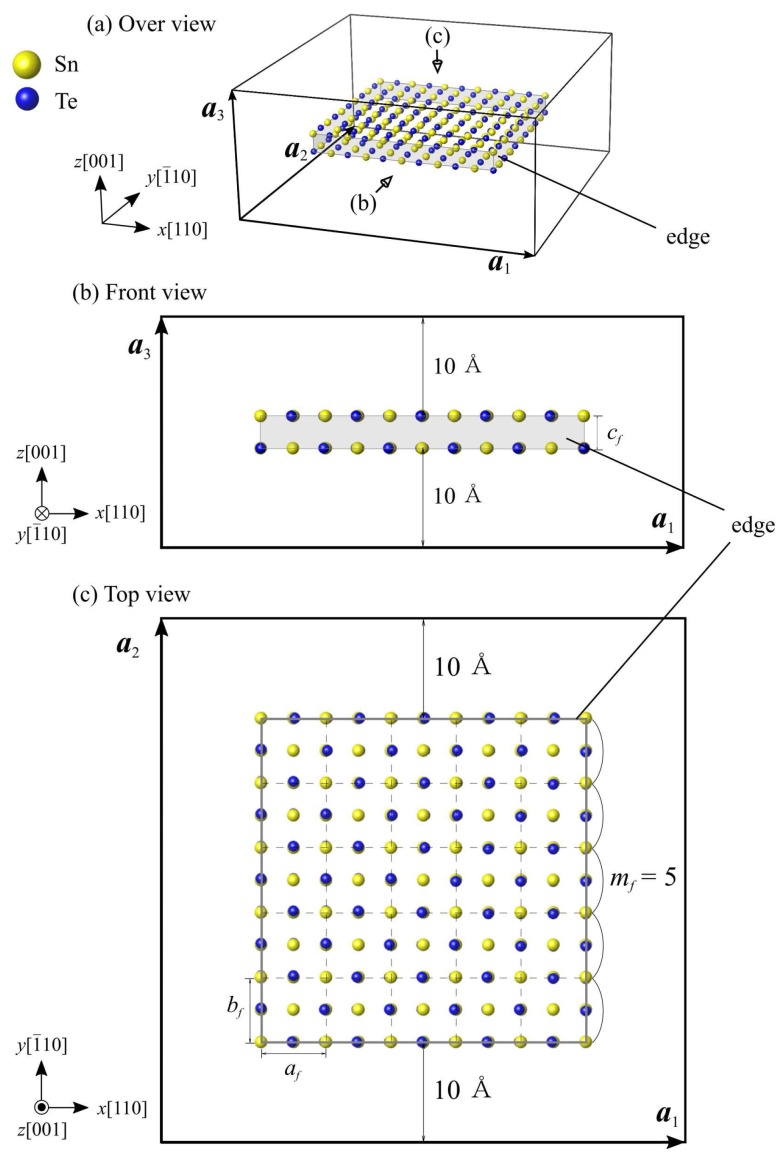
Simulation models of the SnTe nanoflake with a surface area of 5 × 5 unit cells. The gray area and solid lines indicate edges of the SnTe nanoflake. The solid boxes represent the simulation cells. ***a***_1_, ***a***_2_ and ***a***_3_ indicate the simulation cell vectors.

Figure 3 shows the simulation model of SnTe nanoflakes. Here, *m*_f_ denotes the number of unit cells constituting each side of the SnTe nanoflake. To explore the critical ferroelectric size, we calculate several SnTe nanoflakes with *m*_f_ = 1 to 7 (6 to 45 Å) on one side. The model of a SnTe nanoflake with 5 × 5 unit-cells surface area (*m*_f_ = 5) (hereinafter referred to as a 5 × 5 nanoflake) as an example. In this simulation model, there are (2*m*_f_ + 1)^2^ Sn and Te atoms each, for a total of 2 × (2*m*_f_ + 1)^2^ atoms. The three-dimensional periodic boundary condition is applied to the simulation cell. To avoid undesirable interactions from neighboring nanoribbons in image cells, a vacuum region of *l*_v_ = 20 Å is introduced to the *x, y,* and *z* directions of the simulation cell. ***a***_1_, ***a***_2_, and ***a***_3_ in the figure are the simulation cell vectors, and are represented by

***a***_1_ = (*m*_f_*a+ l*_v_, 0, 0), (4)

***a***_2_ = (0, *m*_f_*b + l*_v_, 0), (5)

***a***_3_ = (0, 0, *c + l*_v_), (6)

where, *a*, *b*, and *c* are the equilibrium lattice constants of the SnTe monolayer.

We perform first-principle, density-functional theory (DFT) calculations [22,23]. The effects of nuclei and inner shells are expressed by the project-augmented wave (PAW) method [24,25], and the Sn 4*d*, 5*s*, 5*p* orbitals and the Te 5*s*, 5*p* orbitals are explicitly treated as valence electrons. The electronic wave function is expanded in plane-waves, and the cutoff energy of the plane waves is set to 450 eV. The Brillouin zone integration is performed using a 10 × 1 × 1 Monkhorst-Pack *k*-point mesh for the nanoribbon models and a 1 × 1 × 1 *k*-point mesh for the nanoflake models [26]. The PBE-D3 functional is used for the evaluation of the exchange correlation term [27]. The stable structure is determined by relaxing atomic positions using the conjugate gradient method until the force acting on the atoms became 1.0 × 10^−3^ eV/Å or less. All the first-principles calculations are performed using the Vienna Ab-initio Simulation Package (VASP) code [28,29]. The present calculation condition was confirmed to reproduce the electronic (band structure) and ferroelectric properties of SnTe monolayer via the comparison of experimental data [18].

## 3. Results and Discussion

### 3.1. Ferroelectric Critical Size of SnTe Nanoribbons

**Figure 4:**
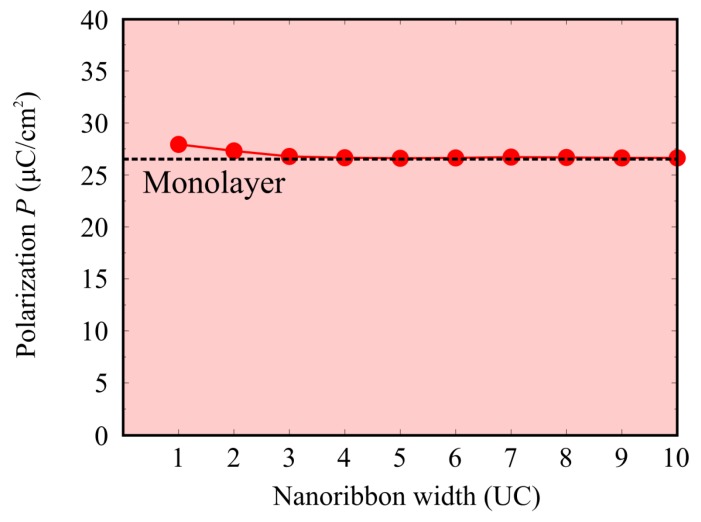
Polarization P in SnTe nanoribbon as a function of the width. The red dotted line shows the ferroelectric polarization of 2D SnTe monolayer. UC denotes the unit cells.

Figure 4 shows the change in the spontaneous polarization *P* of the nanoribbon with respect to the width of the SnTe nanoribbon. The polarization of SnTe nanoribbons are aligned along the longitudinal direction ([110] direction). The black dashed line indicates the polarization value of the SnTe monolayer. Here, the ferroelectric polarization is calculated by the Berry phase approach [30]. Note that, in this study, the indeterminacy of spontaneous polarization via the Berry phase calculations is treated by using the paraelectric phase SnTe structure as the reference (zero-polarization) state. The SnTe nanoribbons with a 10-unit-cells width exhibits the polarization of *P* = 26.6 μC/cm^2^, which is almost the same magnitude as the SnTe monolayer, *P* = 26.5 μC/cm^2^. This indicates that there is no size effect in the nanoribbons with a 10-unit-cell width (65 Å). Even when the nanoribbon width is reduced, the ferroelectric polarization is almost constant at *P* = 26.6 to 27.9 μC/cm^2^, and all simulated nanoribbons show ferroelectric polarization comparable to that of the SnTe monolayer. This means that the SnTe nanoribbon does not exhibit any size-dependence, unlike conventional three-dimensional ferroelectrics such as BaTiO_3_ and PbTiO_3_. In addition, the SnTe nanoribbon with the minimum one-unit-cell width exhibits non-zero ferroelectric polarization. Our result indicates that ferroelectricity does not disappear even in the smallest nanoribbon, and there is, therefore, no critical dimension in which the ferroelectricity disappears in the SnTe nanoribbons.

Conventional three-dimensional crystalline ferroelectrics, such as the perovskite oxides BaTiO_3_ and PbTiO_3_, exhibit remarkable size-dependence, and the ferroelectricity disappears when the material size reaches several nanometers [31,32,33]: for example, the critical ferroelectric size of BaTiO_3_ nano-films was reported to be 2-nm thickness [12,13], that of PbTiO_3_ nano-films was 1.2-nm thickness [14,34], and Pb(Zr,Ti)O_3_ nanodot lost its ferroelectricity when it became 3.2 nm in diameter [10]. Regardless of the material and shape, the ferroelectricity is reduced and finally disappears as the material size decreases. As was explained in the introduction, such size effects and critical dimensions on ferroelectricity originate from two factors: (I) electrostatic effects due to the formation of a depolarization field [16,35,36,37,38], and (II) the reconstruction and rearrangement of atomic and electronic structures due to the low coordination number at the surface (or edge) [36,37,38,39]. 

**Figure 5:**
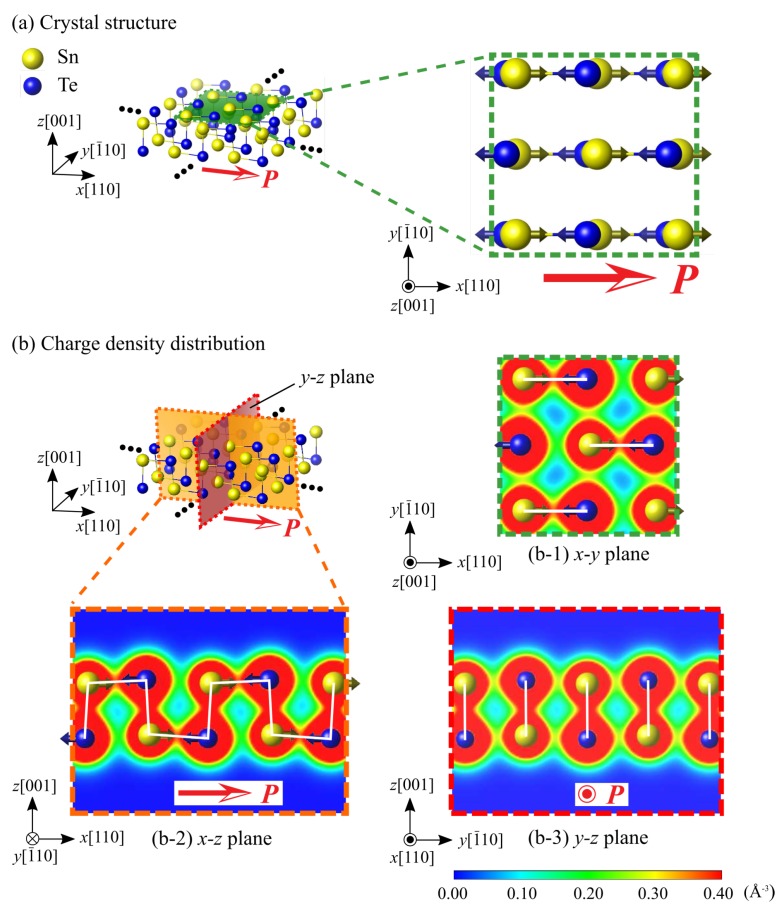
(**a**) Crystal structure of SnTe monolayer and (**b**) charge density distributions in SnTe monolayer. The covalent Sn-Te bonds are shown by white lines. Red arrows *P* indicate the direction of spontaneous polarization in the SnTe monolayer. Yellow and blue arrows indicate the displacement of Sn and Te atoms, respectively.

**Figure 6:**
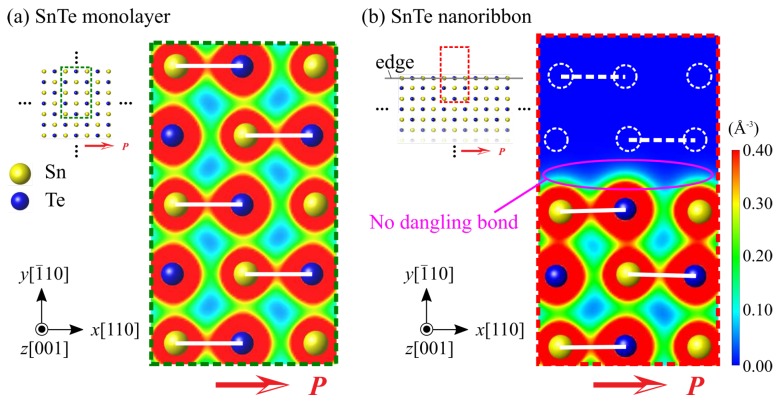
Charge density distributions (**a**) in the SnTe monolayer and (**b**) in the SnTe nanoribbon with 6-unit-cells width. Red arrows *P* indicate the spontaneous polarization. The covalent Sn-Te bonds are shown by white lines. White dotted circles and lines indicate the imaginary Sn or Te atom positions and Sn-Te bonds formed in the SnTe monolayer.

Considering the above discussion on the conventional ferroelectrics, here we investigate the absence of a critical ferroelectric size of SnTe nanoribbons in terms of factors (I) and (II). Considering factor (I), the ferroelectric polarization direction of the SnTe nanoribbon is almost parallel to the edge line, and thereby, no surface polarization charge is induced and no depolarization field is generated. Therefore, the electrostatic factor (I) due to the depolarization field does not occur in SnTe nanoribbons. Next, the dangling bond formation at the edge of the SnTe nanoribbon is examined to consider factor (II). Here, we first refer the bonding structure of the SnTe monolayer (i.e., without dangling bonds) as a reference, as shown in Figure 5. In the SnTe monolayer, spontaneous polarization *P* appears in the [110] direction due to the relative displacement of Sn^2+^ ions in the [110] direction with respect to Te^2−^ ions in the ferroelectric phase (see Figure 5a). Due to the ionic displacement, the SnTe monolayer forms an Sn-Te bond along the [110] direction, which is the same as the direction of spontaneous polarization. This means that the relative displacement and bonding of the Sn^2+^ and Te^2−^ ions in the [110] direction in the SnTe monolayer corresponds to the spontaneous polarization and, thereby, is a characteristic of the ferroelectric manifestation of the SnTe monolayer. Figure 6 compares the bonding situation between in the SnTe monolayer and the SnTe nanoribbon. The white lines in the figure indicate Sn-Te bonds, while the white dashed circles and lines in the SnTe nanoribbon indicate the imaginary Sn or Te position and bond, respectively, which were formed in the SnTe monolayer. As described above, the SnTe monolayer forms an armchair-shaped Sn-Te bond along the [110] direction (see Figure 5b-2), and this situation can be seen alternately appearing on the Sn-Te bond from the top view of the monolayer, as shown in Figure 6a. In general, near the surface or edge, the rearrangement of electrons occurs due to the presence of dangling bonds, which affects ferroelectricity [36,37,38]. On the other hand, focusing on the electron density distribution at the edge of the SnTe nanoribbon in Figure 6b, Sn-Te is also found along the [110] direction, which is almost the same as the electron density distribution of the SnTe monolayer; i.e., the absence of a dangling bond at the edge of SnTe monolayer. This is because the bonding sequence in the SnTe monolayer is mainly along the polar direction of [110], and thereby, the formation of the [110] edge does not introduce any dangling bond. The absence of a dangling bond at the [110] edge in the SnTe nanoribbons makes the ferroelectricity same as that in the SnTe monolayer. Therefore, the absence of factors (I) and (II) leads to the absence of critical ferroelectric size in SnTe nanoribbons.

### 3.2. Ferroelectric Critical Size of SnTe Nanoflakes

**Figure 7:**
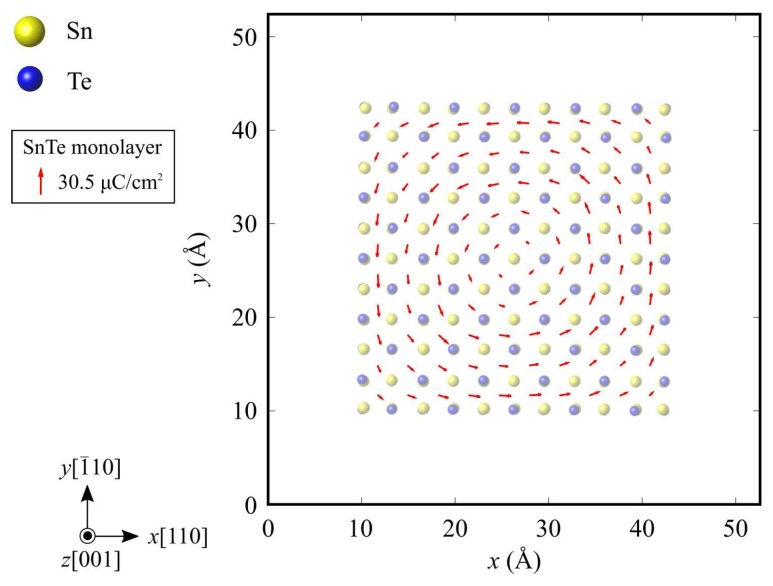
Vector-field representation of local polarization distribution in the 5 × 5 nanoflake. Red arrows indicate the spontaneous polarization.

Figure 7 shows the local polarization distribution in the 5 × 5 nanoflakes. Spontaneous polarization exists and forms a vortex polarization order in the counterclockwise direction. Similar vortex polarization distributions are also observed in the other 6 × 6 and 7 × 7 nanoflakes. Such a polar vortex is characteristic of the polarization order in ferroelectric nanostructures [11,40] because the surface component of polarization is aligned along a surface or edge to prevent the formation of the depolarization field and minimize the electrostatic energy efficiently. Since SnTe nanoflakes are surrounded by edges on all sides, a surface polarization charge is induced at the edges, and a depolarization field is generated inside the SnTe nanoflakes. Since the parallel polarization to the edge of the nanoflake does not produce any surface polarization charge or depolarization field, the formation of vortex polarization is more energetically stable than the original straight form of ferroelectric polarization. 

To evaluate the critical dimension of the ferroelectricity in SnTe nanoflakes with vortex polarization, here we consider the toroidal moment ***G***. The toroidal moment ***G*** is used as a physical quantity that characterizes vortex polarization appearing in nanoscale ferroelectric materials [10,41,42], and is given by the following equation [42]

(7) $$G=\frac{1}{2N}\sum_{k}r_{k}\times P_{k}$$

where **r***_k_* is the position vector of the *k*-th local unit cell, ***P****_k_* is the local spontaneous polarization at position **r***_k_*, and *N* is the number of local unit cells included in the simulation cell. The sum is taken of all unit cells in the simulation cell. The local polarization is evaluated using the Born effective charge tensors [30]. The site-by-site local polarization can be calculated by

(8) $$P_{i}=\frac{e}{\Omega_{c}}w_{j}Z_{j}u_{j}$$

where Ω*_c_* is the volume of the local unit cell *i*; *e* and ***u****_j_* denote the electron charge and the atomic displacement vector relative to the ideal lattice site (paraelectric lattice site) of atom *j*, respectively. The index *j* covers all atoms in the local unit cell *i*. ***Z****j* is the Born effective charge tensor of atom *j* and *wj* is a weight factor.

**Figure 8:**
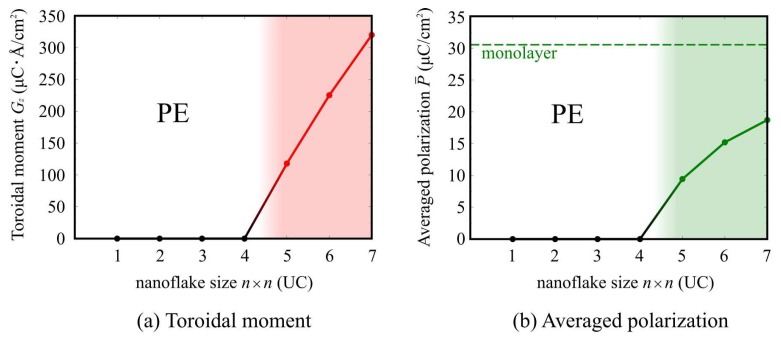
(**a**) Toroidal moment, ***G***, and (**b**) averaged polarization, ***P***, in SnTe nanoflake as a function of SnTe nanoflake size. The green dotted line indicates the polarization of the SnTe monolayer. PE indicates the paraelectric phase.

Figure 8 shows the toroidal moment *G_z_* and the average polarization *P* for each SnTe nanoflake size. Note that we show the *z* component of toroidal moment ***G*** because all of the vortex polarization appears on the *xy* plane. The toroidal moment *G_z_* decreases as the size of the SnTe nanoflakes decreases. When the size of the SnTe nanoflakes become 4 × 4 unit-cell size or less, the toroidal moment *G_z_* becomes zero. Here, we also show the averaged polarization in panel (b), as defined by

(9) $$\bar{P}=\frac{1}{N}\sum_{k}\left|P_{k}\right|$$

Such size-dependent behavior is also seen in the averaged polarization. The average polarization value decreases as the size of the SnTe nanoflakes decreases, and the polarization becomes 0 when one side is less than four unit cells (25 Å). These results indicate that, in contrast to the SnTe monolayer (2D) and nanoribbons (1D), the SnTe nanoflakes (0D) exhibit remarkable size-dependence and a critical dimension at which ferroelectricity disappears. The critical dimension is evaluated to be four unit cells on one side (about 25 Å). This suggests that structural low-dimensionality can affect the ferroelectricity of SnTe system and lead to the appearance of a ferroelectric critical size.

**Figure 9:**
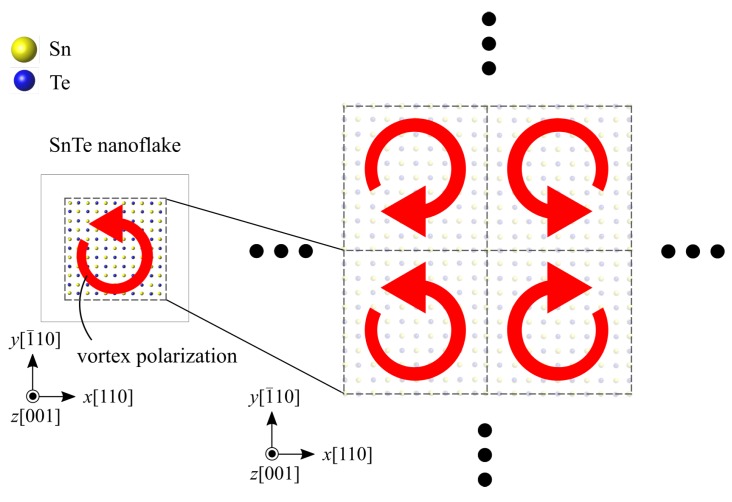
Simulation model of the edge-free SnTe with periodic polarization vortices. Red arrows indicate the direction of vortex polarization.

**Figure 10:**
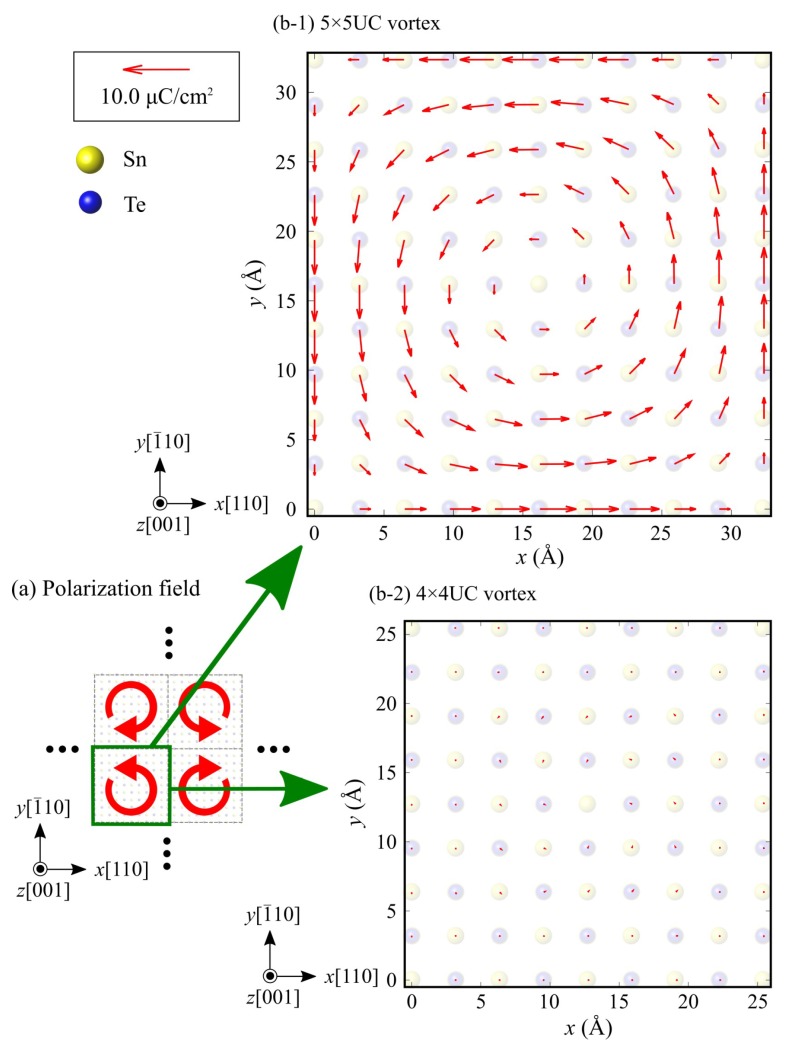
Vector-field representation of the spontaneous polarization of the imaginary edge-free SnTe models with a polarization-vortex periodicity of (**a**) 5 × 5 and (**b**) 4 × 4 unit cells. Red arrows indicate spontaneous polarization.

In the following, we discuss the appearance of critical dimension of the vortex polarization in the SnTe nanoflakes. As discussed in Section 3.1, the factors that cause the size effect and critical dimension appear are: (i) the electrostatic effect, due to the formation of a depolarization field [16,35], and (ii) the reconstruction and rearrangement of the atomic and electronic structure due to a lower coordination number at a surface or edge [36,37,38]. In order to examine the effect of these factors, we calculate an imaginary model of an edge-free SnTe monolayer with a vortex polarization that is the same as the nanoflakes, as shown in Figure 9, and compare the results. This model consists of periodically arranged clockwise and counterclockwise polarization vortices in a SnTe monolayer, and each polarization vortex mimics a nanoflake with a vortex polarization but without any edge structures. Since this imaginary edge-free SnTe monolayer model is free from the edge and the resulting (coinciding) electrostatic depolarization field and dangling bonds, through comparison between the SnTe nanoflakes and this imaginary edge-free model, one can extract how the existence of edge and electrostatic field and/or dangling bonds affect the ferroelectricity of the SnTe nanoflakes. Figure 10 shows the calculated local polarization field of the imaginary edge-free SnTe models with polarization-vortex periodicity of 5 × 5 and 4 × 4 unit cells. The edge-free SnTe model with a 5 × 5 unit cell size exhibits a quasi-stable vortex polarization, as shown in Figure 10a, which is almost same as that observed in the 5 × 5 SnTe nanoflake, as shown in Figure 7. On the other hand, no spontaneous polarization is observed in the edge-free SnTe model with a 4 × 4 unit cell or less (Figure 10b). This is also consistent with the absence of polarization and paraelectric nature of the 4 × 4 SnTe nanoflake. These results indicate that the presence or absence of edges does not affect the appearance of a ferroelectric critical size of SnTe nanoflakes. Therefore, the effects of factors (i) the depolarization field and (ii) the dangling formation at the edges are not the primary causes of the disappearance of the vortex polarization in the SnTe nanoflakes. The situation of the SnTe nanoflakes is clearly different from that of the conventional ferroelectrics, where the critical size appears due to these two factors.

**Figure 11:**
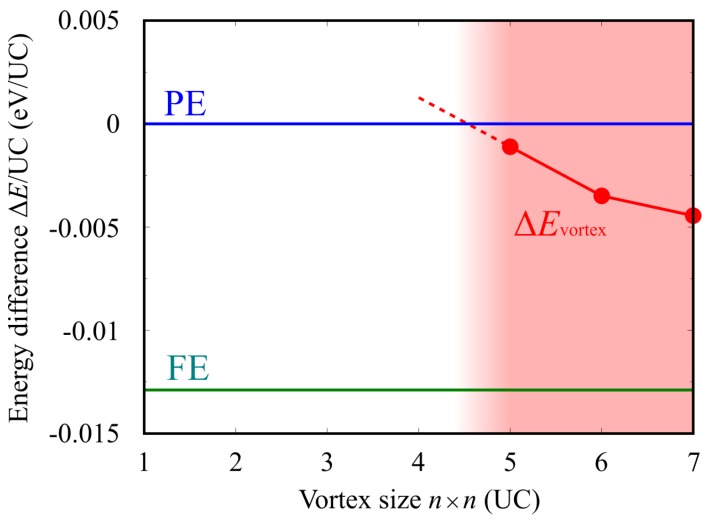
Total energy difference of the vortex polarization Δ*E*_vortex_ and the ferroelectric phase of the SnTe monolayer on the basis of the paraelectric phase as a function of the size of vortex polarization.

From the above discussion, there is the possibility that the critical size of SnTe nanoflakes is not due to the presence of edges, but the intrinsic size dependence of the vortex polarization itself. To confirm this possibility, we investigate the energetics of the edge-free SnTe models with different vortex sizes. Again, this model is free from the edge and can only extract the effect of the size of vortex polarization. Figure 11 shows the total energy difference of the polar vortex phase from the total energy of the paraelectric (PE) phase ∆*E*_vortex_ as a function of periodic vortex size. For comparison, the ferroelectric (FE) phase with straight polarization (single domain) is also shown in Figure 11. Here, the total energy difference ∆*E*_vortex_ is normalized by dividing by the number of unit cells in each system. ∆*E*_vortex_ is negative at 7 × 7 vortex size, and the vortex size is more stable than the paraelectric phase. ∆*E*_vortex_ increases with decreasing vortex polarization size, and, finally, ∆*E*_vortex_ may reach the energy of the PE phase at the 4 × 4 vortex size or less. As shown in Figure 10b-2, the 4 × 4 vortex model becomes paraelectric, and thus the total energy of 4 × 4 size or less is same as that of the PE phase. This indicates that the vortex polarization increases its total energy as the vortex size decreases, and finally the vortex polarization with a smaller than 5 × 5 size becomes more energetically unstable than the PE phase and the vortex polarization cannot be formed. We thus confirm that the vortex form of polarization intrinsically exhibits the size dependence, and there exists a critical size of vortex polarization itself. Therefore, the ferroelectric critical size observed in the SnTe nanoflakes originates from the intrinsic size limit of polarization vortices. 

**Figure 12:**
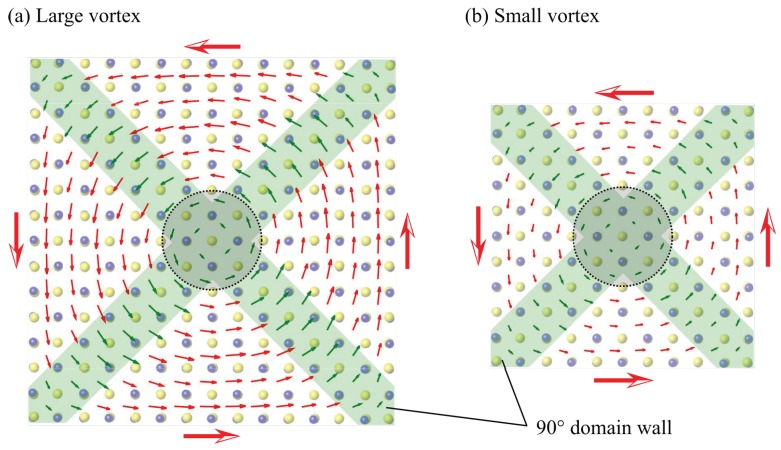
Schematic illustration of domain structure in the SnTe nanoflake consisting of 90° domain walls. Red arrows indicate spontaneous polarization and green areas indicate domain walls.

The increase in the total energy of vortex polarization due to the decrease in the size of vortices is due to the increase in domain wall densities. As shown in Figure 12, the vortex structure has four domains (white area), and they are separated by four 90° domain walls (green area). As the size of the vortex polarization decreases, the ratio of the domain wall per unit surface area increases, and the total energy increases. Such a high density of domain walls in the smaller vortex polarization is the primary cause of the loss of polarization in the smaller SnTe nanoflakes.

With the recent advance in manufacturing technology for two-dimensional materials, such as graphene, there are numerous experimental studies which reported the fabrication of various nanostructures of 2D materials, including graphene nanoribbons, nanoflakes, nanotubes and nanohorns. Using the fabrication techniques of graphene and its nanostructures, the fabrication of SnTe nanoribbons and nanoflakes presented in this study should be experimentally feasible. Thus, our results may stimulate an experimental study to fabricate and characterize the unique ferroelectric properties of SnTe monolayer and nanostructures. 

## 4. Conclusions

In this study, we investigated a ferroelectric critical size of low-dimensional SnTe nanostructures such as nanoribbons (1D) and nanoflakes (0D) using first-principle density-functional theory calculations. We demonstrated that the smallest (one-unit-cell width) SnTe nanoribbon could sustain ferroelectricity and there was no ferroelectric critical size in the SnTe nanoribbons. On the other hand, the SnTe nanoflakes formed a vortex of polarization and lost its toroidal ferroelectricity below the surface area of 4 × 4 unit cells (about 25 Å on one side). We also revealed the atomic and electronic mechanism of the absence or presence of critical size in SnTe low-dimensional nanostructures. Our result provides an insight into intrinsic ferroelectric critical sizes for low-dimensional chalcogenide layered materials.

## Figures and Tables

**Table 1:** Calculated edge formation energy *E*_edge_ of [110] and [100] edge in layered SnTe.

Edge Direction	[110]	[100]
*E*_edge_ (eV/Å)	0.097	0.127

## References

[1] Ahn C.H., Rabe K.M., Triscone J.M. (2004). Ferroelectricity at the nanoscale: Local polarization in oxide thin films and heterostructures. Science.

[2] Gruverman A., Kholkin A. (2005). Nanoscale ferroelectrics: Processing, characterization and future trends. Rep. Prog. Phys..

[3] Scott J. (2007). Applications of modern ferroelectrics. Science.

[4] Wasa K., Haneda Y., Sato T., Adachi H., Setsune K. (1998). Crystal growth of epitaxially grown PbTiO_3_ thin films on miscut SrTiO_3_ substrate. Vacuum.

[5] Fujisawa H., Shimizu M., Niu H., Nonomura H., Honda K. (2005). Ferroelectricity and local currents in epitaxial 5-and 9-nm-thick Pb(Zr, Ti)O_3_ ultrathin films by scanning probe microscopy. Appl. Phys. Lett..

[6] Gu H., Hu Y., You J., Hu Z., Yuan Y., Zhang T. (2007). Characterization of single-crystalline PbTiO_3_ nanowire growth via surfactant-free hydrothermal method. J. Appl. Phys..

[7] Yun W.S., Urban J.J., Gu Q., Park H. (2002). Ferroelectric properties of individual barium titanate nanowires investigated by scanned probe microscopy. Nano Lett..

[8] Wang W., Varghese O.K., Paulose M., Grimes C.A., Wang Q., Dickey E.C. (2004). A study on the growth and structure of titania nanotubes. J. Mater. Res..

[9] Morrison F.D., Ramsay L., Scott J.F. (2003). High aspect ratio piezoelectric strontium-bismuth-tantalate nanotubes. J. Phys. Condens. Matter.

[10] Naumov I.I., Bellaiche L., Fu H. (2004). Unusual phase transitions in ferroelectric nanodisks and nanorods. Nature.

[11] Schilling A., Byrne D., Catalan G., Webber K., Genenko Y., Wu G., Scott J., Gregg J. (2009). Domains in ferroelectric nanodots. Nano Lett..

[12] Fong D.D., Stephenson G.B., Streiffer S.K., Eastman J.A., Auciello O., Fuoss P.H., Thompson C. (2004). Ferroelectricity in ultrathin perovskite films. Science.

[13] Junquera J., Ghosez P. (2003). Critical thickness for ferroelectricity in perovskite ultrathin films. Nature.

[14] Despont L., Koitzsch C., Clerc F., Garnier M., Aebi P., Lichtensteiger C., Triscone J.M., de Abajo F.G., Bousquet E., Ghosez P. (2006). Direct evidence for ferroelectric polar distortion in ultrathin lead titanate perovskite films. Phys. Rev. B.

[15] Shimada T., Wang X., Kondo Y., Kitamura T. (2012). Absence of ferroelectric critical size in ultrathin PbTiO_3_ nanotubes: A density-functional theory study. Phys. Rev. Lett..

[16] Mehta R., Silverman B., Jacobs J. (1973). Depolarization fields in thin ferroelectric films. J. Appl. Phys..

[17] Cochran W. (1960). Crystal stability and the theory of ferroelectricity. Adv. Phys..

[18] Chang K., Liu J., Lin H., Wang N., Zhao K., Zhang A., Jin F., Zhong Y., Hu X., Duan W. (2016). Discovery of robust in-plane ferroelectricbloity in atomic-thick SnTe. Science.

[19] Chang K., Kaloni T.P., Lin H., Bedoya-Pinto A., Pandeya A.K., Kostanovskiy I., Zhao K., Zhong Y., Hu X., Xue Q.-K. (2019). Enhanced spontaneous polarization in ultrathin SnTe films with layered antipolar structure. Adv. Mater..

[20] Chang K., Parkin S.S. (2019). The growth and phase distribution of ultrathin SnTe on graphene. APL Mater..

[21] Liu K., Lu J., Picozzi S., Bellaiche L., Xiang H. (2018). Intrinsic origin of enhancement of ferroelectricity in SnTe ultrathin films. Phys. Rev. B.

[22] Hohenberg P., Kohn W. (1964). Inhomogeneous electron gas. Phys. Rev..

[23] Kohn W., Sham L.J. (1965). Self-consistent equations including exchange and correlation effects. Phys. Rev..

[24] Blöchl P.E. (1994). Projector augmented-wave method. Phys. Rev. B.

[25] Kresse G., Joubert D. (1999). From ultrasoft pseudopotentials to the projector augmented-wave method. Phys. Rev. B.

[26] Monkhorst H.J., Pack J.D. (1976). Special points for Brillouin-zone integrations. Phys. Rev. B.

[27] Grimme S., Antony J., Ehrlich S., Krieg H. (2010). A consistent and accurate ab initio parametrization of density functional dispersion correction DFT-D for the 94 elements H-Pu. J. Chem. Phys..

[28] Kresse G., Hafner J. (1993). Ab initio molecular dynamics for liquid metals. Phys. Rev. B.

[29] Kresse G., Furthmüller J. (1996). Efficient iterative schemes for ab initio total-energy calculations using a plane-wave basis set. Phys. Rev. B.

[30] Meyer B., Vanderbilt D. (2002). Ab initio study of ferroelectric domain walls in PbTiO_3_. Phys. Rev. B.

[31] Zhang Y., Li G.-P., Shimada T., Wang J., Kitamura T. (2014). Disappearance of ferroelectric critical thickness in epitaxial ultrathin BaZrO_3_ films. Phys. Rev. B.

[32] Hong J., Fang D. (2008). Size-dependent ferroelectric behaviors of BaTiO_3_ nanowires. Appl. Phys. Lett..

[33] Polking M.J., Han M.-G., Yourdkhani A., Petkov V., Kisielowski C.F., Volkov V.V., Zhu Y., Caruntu G., Alivisatos A.P., Ramesh R. (2012). Ferroelectric order in individual nanometre-scale crystals. Nat. Mater..

[34] Béa H., Fusil S., Bouzehouane K., Bibes M., Sirena M., Herranz G., Jacquet E., Contour J.-P., Barthélémy A. (2006). Ferroelectricity down to at least 2 nm in multiferroic BiFeO_3_ epitaxial thin films. Jpn. J. Appl. Phys..

[35] Batra I.P., Silverman B. (1972). Thermodynamic stability of thin ferroelectric films. Solid State Commun..

[36] Meyer B., Padilla J., Vanderbilt D. (1999). Theory of PbTiO_3_, BaTiO_3_, and SrTiO_3_ surfaces. Faraday Discuss..

[37] Bungaro C., Rabe K. (2005). Coexistence of antiferrodistortive and ferroelectric distortions at the PbTiO_3_ (001) surface. Phys. Rev. B.

[38] Umeno Y., Shimada T., Kitamura T., Elsässer C. (2006). Ab initio density functional theory study of strain effects on ferroelectricity at PbTiO_3_ surfaces. Phys. Rev. B.

[39] Shimada T., Tomoda S., Kitamura T. (2009). Ab initio study of ferroelectricity in edged PbTiO_3_ nanowires under axial tension. Phys. Rev. B.

[40] Wang X., Yan Y., Shimada T., Wang J., Kitamura T. (2018). Ferroelectric critical size and vortex domain structures of PbTiO_3_ nanodots: A density functional theory study. J. Appl. Phys..

[41] Shimada T., Wang X., Tomoda S., Marton P., Elsässer C., Kitamura T. (2011). Coexistence of rectilinear and vortex polarizations at twist boundaries in ferroelectric PbTiO_3_ from first principles. Phys. Rev. B.

[42] Pilania G., Alpay S., Ramprasad R. (2009). Ab initio study of ferroelectricity in BaTiO_3_ nanowires. Phys. Rev. B.

